# A telemonitoring system to support CPAP therapy in patients with obstructive sleep apnea: a participatory approach in analysis, design, and evaluation

**DOI:** 10.1186/s12911-022-01912-8

**Published:** 2022-06-26

**Authors:** Shokoufeh Aalaei, Mahnaz Amini, Mohammad Reza Mazaheri Habibi, Hadi Shahraki, Saeid Eslami

**Affiliations:** 1 Department of Medical Informatics, Faculty of Medicine, Mashhad University of Medical Sciences, Mashhad, Iran; 2 Faculty of Medicine, Lung Diseases Research Center, Mashhad University of Medical Sciences, Mashhad, Iran; 3 Department of Health Information Technology, Varastegan Institute for Medical Sciences, Mashhad, Iran; 4 Department of Computer Engineering, Faculty of Industry and Mining, University of Sistan and Baluchestan, Zahedan, Iran; 5 Department of Medical Informatics, University of Amsterdam, Amsterdam, the Netherlands; 6 Pharmaceutical Research Center, School of Pharmacy, Mashhad University of Medical Sciences, Mashhad, Iran

**Keywords:** Mobile health, Telemedicine, Sleep apnea, Continuous positive airway pressure, Patient education, Adherence

## Abstract

**Background:**

Continues positive airway pressure (CPAP) therapy is a gold standard treatment for moderate to severe cases of OSA (obstructive sleep apnea). The present research aimed to describe the analysis, design, and evaluation of a telemonitoring system to improve CPAP adherence in patients afflicted with OSA.

**Methods:**

The telemonitoring system was developed in five phases. In the exploratory phase, the body of related literature was reviewed. Then a need analysis was conducted through a focus group discussion with sleep medicine specialists and sales company representatives and an interview with patients. The third phase involved data integration. Then the content and system development were done based on the previous phases. Finally, usability and functionality tests were used to evaluate the system.

**Results:**

The exploratory phase and the needs analysis were conducted by four sleep medicine specialists, two medical informatics specialists, six key figures of the sales companies, two system developers, and 46 patients in different phases. Based on the results obtained from the data integration phase, the telemonitoring system involved three main parts: a patient’s application, a doctor’s portal, a selling companies’ portal (operator’s portal) along with facilitating software for patients to send the CPAP data. Usability and functionality tests were given to 7 and 10 patients, respectively. The total number of usability issues reported by users in the evaluation process was 18, with an average of 2.5 issues per user. The installation problems, disrupted links and improper playing of videos were the main functionalities problems that were solved.

**Conclusion:**

The telemonitoring system, as a means of communication between patients, doctors, and selling companies, can be used to support patients clinically and technically. It has the potential to improve CPAP adherence in patients with OSA.

## Highlights

- The study developed and evaluated a telemonitoring system to support CPAP therapy in patients with obstructive sleep apnea.
- Participatory approach in analysis, design and evaluation increased the potential effectiveness of the telemonitoring system.
- Using different data collection methods can guarantee meeting all requirements and make using the existing capabilities in the best way.
- Using the behavior change techniques in preparing content and system capabilities probably enhances the changing patient’s behavior in using the CPAP device.
- Using different system evaluation methods can help identify system defects and correct them in further research.

## Background

Obstructive sleep apnea (OSA) is the most common sleep disorder caused by temporary obstruction of the upper airway during sleep. As the findings reported by Benjafield et al. (2019) showed, an overall number of 936 million adults aged 30–69 years indicated mild to severe obstructive sleep apnea (AHI ≥ 5 events/hour). Besides, 425 million adults whose ages ranged between 30 and 69 years had a moderate to severe level of obstructive sleep apnea (AHI ≥ 15 events/hour) on a global scale [1]. This disease negatively influences the patients’ quality of sleep. It further adversely affects arterial oxygen and can lead to disruption of daily routines, deadly accidents, metabolic syndrome, cardiovascular diseases, and even death caused by a heart attack or cerebral stroke. A timely diagnosis and treatment of sleep apnea can reduce the morbidity and mortality rate [2–4].

Continuous positive airway pressure (CPAP) is the gold standard treatment for moderate to severe cases of OSA. CPAP therapy is only effective when the CPAP device is used regularly and for a long time [5]. Yet, patients’ adherence to its regular use has shown to be poor. The non-adherence rate is 34.1%, which reduces the therapeutic effect [6, 7]. It seems that with the advent of new telecommunication technologies, it is time to move towards patient-centered interventions that provide remote monitoring, usually via telemedicine.

It has proved that telemedicine is effective in the management of sleep disorders [8]. The data recorded by the CPAP device (e.g., the pressure of the device, mask leakage, number of apnea, etc.) are easily transferred to the healthcare center and the pressure of the device is easily adjusted from a distance. Therefore, telemedicine can help a complete evaluation of real adherence to CPAP. A body of research showed that educating patients on OSA and using CPAP and providing feedback can be done through communication channels such as videoconferencing, mobile applications, and websites [9–13].

On the one hand, patients with OSA that undergo CPAP therapy require constant clinical and technical support for adherence to medical orders. In such circumstances, telemedicine has the potential to empower and improve self-care in patients with chronic diseases, including OSA. Thus, we require appropriate tools that can provide telemedicine services for patients. Accordingly, the present research aims to describe the analysis, design, and evaluation of a telemonitoring system for patients afflicted with OSA, who undergo CPAP therapy.

## Methods

### Design and setting

The present research is a qualitative study using a participatory approach to engage all stakeholders including patients with OSA who used CPAP machines, sleep medicine specialists, sales companies, researchers, and software developers, in different steps of need analysis, design, and evaluation process.

The study was conducted in Mashhad, the second metropolis of Iran. All sleep medicine specialists who participated in this study were faculty members of Mashhad University of Medical Sciences. The patients were selected in two ways: those who had purchased their CPAP device from Resmed or Weinmann companies in Mashhad and those who were referred to the specialists mentioned above to treat sleep apnea and for follow-up purposes.

**Fig. 1:**
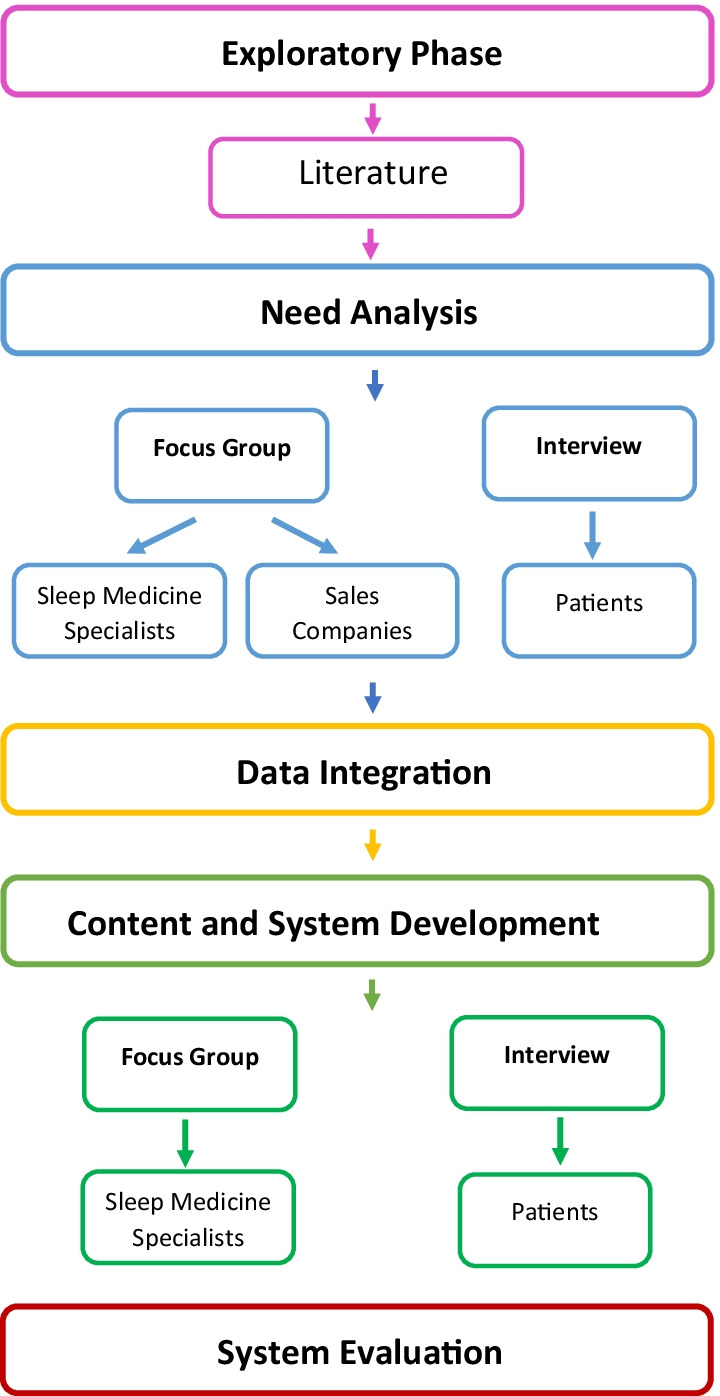
Overview of the development and evaluation process

The present research was conducted in five phases. The first was exploratory and investigated the body of related literature. In the second phase, a need analysis was done with the cooperation of the main stakeholders. The third phase involved data integration. The fourth phase involved content development and the design of a telemonitoring system named Roya. The fifth phase involved the evaluation of the content and system. Figure 1 summarizes this procedure.

### Exploratory phase

The body of related literature on sleep apnea treatment and a focus on patient’s adherence to CPAP belonged to either of the following two categories:

- Studies with a focus on effective factors for adherence to CPAP
- Studies implementing an intervention to increase adherence to CPAP

#### Exploring effective factors on adherence to CPAP

In the body of related literature, among the effective factors of adherence to CPAP were patients’ characteristics, the severity of disease, side effects of using the device, method of conducting the sleep test and titration, claustrophobia, inadequate support of the clinical team, lacking cooperation of the spouse, inadequate knowledge and high costs of treatment and equipment. These were among the main barriers to adherence to treatment [7, 14–18]. Among the facilitators of adherence to CPAP were: the willingness to rid of symptoms and a positive attitude to treatment, awareness of the adverse effects of the disease, fear of the social consequences, disrupting others’ sleep, trust in healthcare providers, spouse’s cooperation, feeling physically improved, use of a humidifier, changing the design of the device, benefiting of social, clinical and behavioral change supports [7, 14–18].

#### Exploring interventions for adherence to CPAP

According to a review by Askland et al. (2020) published by Cochrane [19], interventions in the literature can be divided into three types, educational, supportive and behavioral.

*Educational interventions* These interventions provide general information about OSA and CPAP therapy through different techniques including educational videos, group sessions, personalized explanation of polysomnography (PSG) reports, and positive/negative risk message framing [20–23]. Although educating patients is the primary way of involving them in the therapeutic process, it has a minor effect on CPAP adherence as a complicated behavior [18]. So, it is suggested the educational interventions be accompanied by other interventions.

*Supportive interventions* These interventions provide participants with further clinical follow-up by the clinical staff through telemonitoring to remove barriers or difficulties in using CPAP. The principal privilege of this type of intervention is that patients are encouraged to provide regular feedback on the experience of using the device. Therefore, the therapeutic barriers and problems are removed at the right time. Telemonitoring services in different formats and platforms, such as peer support, web-based, and personalized programs are among supportive interventions [24–30].

*Behavioral interventions *These include interventions with psychotherapeutic techniques based on behavioral, cognitive, or models related to health behavior change. Behavioral interventions addressed modifiable and measurable constructs that influenced the health beliefs about OSA and CPAP therapy and adherence to CPAP behavior. In different studies, various motivational strategies such as Motivational Enhancement Therapy (MET), Socio-Cognitive Theory (SCT), and habit formation audiotapes [31–34] were mixed with educational and supportive materials to enhance effectiveness [35–39].

### Need analysis

In the second phase, a need analysis was done with the cooperation of the main stakeholders. The purpose was to gain an in-depth understanding of the services needed by patients. So, after each meeting, the key concepts were extracted and presented at the next session to explore more.

#### Meeting with sleep medicine specialists

To better understand the requirements to provide telemedicine services for OSA patients who use CPAP, different meetings were held with a panel of sleep medicine specialists (n = 4) and medical informatics specialists (n = 2). In these meetings, patients’ needs and current follow-up plans by physicians and selling companies were investigated.

#### Meeting with key figures of the device selling companies

These meetings were independently held with the key figures of the Resmed (n = 4) and Weinmann (n = 2) companies. In these meetings, services provided to patients such as follow-up plans and instructional materials, and technical capabilities and limitations of devices were discussed.

#### Interview with patients

To know the needs of patients who use CPAP, semi-structured in-depth interviews were held with 29 patients who did not adhere to the treatment. The detail is described in our previous paper [7].

### Data integration

According to the literature review, multiple meetings with sleep specialists and key figures of the device selling companies, and patients’ interviews, the following topics were extracted:

1. Use of different architectures for the telemonitoring system in other countries and further investigations to design and implement the new system in Iran considering the existing technical limitations,
2. Physicians’ concerns about the independence of the device selling companies in the patients’ follow-up and the lack of continuous communication with healthcare providers,
3. Companies’ emphasis and willingness to involve the clinical team in patient follow-up and provision of clinical support,
4. Technical issues such as the absence of a data sending option in CPAP device in a wireless mode to the central system,
5. Barriers to CPAP adherence such as patients’ inadequate knowledge and patients problems in using CPAP device,
6. Use of a combination of different interventions (educational, supportive, and behavioral) to increase effectiveness,
7. Apply behavior change theories and techniques to influence the health beliefs about OSA and CPAP therapy and adherence to CPAP behavior.

### System and content development

#### System architecture

Based on topics 1, 2, 3, and 4, the required parts for designing the telemonitoring system were recognized. This system had to be designed in a way to communicate with different groups (e.g., doctors as the clinical service providers, the device selling companies as the technical service providers, and patients as the service recipients). Also, certain facilities had to be provided for patients to send the CPAP data. Thus, three main parts were proposed including the patient’s application, the doctor’s portal, and the operator’s portal (selling company’s portal) along with facilitating software for patients to send the CPAP data.

Topics 5, 6, and 7 led us to provide clinical, educational, and behavioral supports for patients, simultaneously.

- *Clinical support* involves medical support by doctors such as advice on mask replacement, change of pressure, the answers to medical queries, and other instances of medical support.
- *Educational support* refers to information provided to raise patients’ awareness of apnea and CPAP therapy.
- *Behavior change support* refers to measures taken specifically to change a patient’s way of thinking or acting based on a behavior change model or theory to increase the rate of using CPAP eventually.

Clinical support was provided by monitoring patients’ status and sending appropriate feedback and recommendations through the doctor’s portal. Also, to provide education and behavioral support, the applicable content was made available through the patient’s application. It is noteworthy that some of the information displayed on the doctor’s portal and the patient’s application was fed by data in the operator’s portal. The details are described in the result section.

#### Content development and evaluation

To provide content for the patient’s application, considering the extracted topics and previous findings [40–43], the socio-cognitive theory (SCT) was selected as the basis of content development, which has four constituent constructs: knowledge, perceived risk, outcome expectancy, and self-efficacy.

Perceived risk has to do with the patient’s perceived susceptibility to health threats. In other words, perceiving that untreated OSA is followed by adverse effects. Outcome expectancy refers to the potential outcomes of using or not using CPAP and the possible impact of using CPAP on reducing the associated risks. Self-efficacy implies one’s perceived capability of showing a particular behavior. In other words, it involves perceiving oneself as capable of using the CPAP device regularly under any condition.

Considering socio-cognitive theory and behavior change techniques [54, 55], sleep apnea-related (the definition, symptoms, risk factors, and negative consequences of sleep apnea) and CPAP-related content (benefits and the probable side effects of using CPAP, equipment description, cleaning and replacement methods, troubleshooting, and how to travel with CPAP) were taken as the major portion of the content. This content was derived from credible global websites, existing publications, and sleep specialists’ opinions. Table 1 shows the relationship between the behavior change techniques and the prepared content.

**Table 1:** Behavior change techniques, definition, and content of the application

Techniques used	Definition	Content
Provision of general information	General information about the behavioral risks and unfavorable consequences of misbehaviors This technique is used to raise patients’ awareness of the perceived risk of the disease	Definition of disease (basics and facts) Symptoms of apnea Risk factors of apnea Short explanation of the diagnosis and treatment procedures Healthy sleep habits PAP therapy (treatment with positive air pressure)
Provision of information about consequences	Information about the benefits, costs and outcomes of showing or not showing a certain behavior	Consequences of apnea Benefits of using CPAP in preventing diseases and accidents Benefits of using CPAP for health and well-being Adverse effects of using the device
Encouragement to act as required	Encouraging someone to decide to act (behavior change) or set a goal to access behavior change	Score of the extent to which the device is used
Barrier identification	Identifying the barriers to showing a certain behavior and planning to overcome them	Side effects and how to avoid them List of non-adherent patients’ problems and solutions
Destined action	Acknowledging or rewarding someone for the attempts and performance	Feedback message
Task and duty setting	Setting small tasks and duties to change a behavior and increasing the difficulty level to reach the desired behavior	Issues with using the device (the patient is provided with recommendations to get used to the device gradually)
Giving instructions	Giving instructions on planning and implementing desired healthy behaviors	Side effects of using CPAP and how to solve them Introducing the equipment used Replacing the equipment Cleaning the equipment Troubleshooting and solving issues with the device How to use the device while travelling
Behavior modeling or showing	Displaying how to behave appropriately to a patient by a specialist	Making videos to include: Reduced volume of device Increased ease of mask Mask maintenance Solving the mask leakage problem Cleaning the equipment
Feedback provision	Providing feedback to someone on the recorded behavior and evaluating it against a set of standards or others’ performance	A report on performance within the past week A report on overall performance Comparison of actual and expected performance based on standards

The accuracy, comprehensibility, clarity, and simplicity of the content were assessed by four sleep medicine specialists in a focus group and finalized. Also, a sample of target patients evaluated the difficulty of phrases to decrease the cases of ambiguity and erroneous inferences made of the utterances or the meanings. If a problem was found, the content was changed as required.

### System evaluation

#### Usability testing of patient’s application

To test the usability of the patient’s application, a think-aloud protocol was used, which is a user-based and empirical method based on the observation of system performance in time [44]. The think-aloud method collects data about users’ cognitive growth in working with the system. In this method, users are asked to state out loud whatever they see, think about, and feel, as well as the questions that arise in their minds or their decisions.

For this purpose, seven patients who used CPAP were selected. Two trained facilitators and a software engineer were also present during the evaluation process. The evaluation process was followed in a room of a sleep test clinic within a peaceful and light environment. To conduct the evaluation process, a scenario was developed based on the tasks and duties of the Roya telemonitoring system. All users’ interaction with the system and their voices were recorded by a screen recorder on a tablet computer and a microphone. When the evaluations were completed, all the recorded documents were analyzed by the facilitators. To categorize problems, the method suggested by Haak et al. was used, according to which the problems were categorized into four groups, including system layout, terminology, data entry, and comprehensiveness.

The detail of the usability evaluation process is provided in Appendix 1A.

#### Functionality testing of patient’s application:

In this phase, the primary purpose of functionality testing was to check the success of:

- installing the application on smartphones, each with a different version of the Android operating system,
- transferring the CPAP data from the operator’s portal to the patient’s application,
- transferring responses to daily questions and user problems from the application to the doctor’s portal,
- displaying charts and content in different mobile sets,
- showing videos integrated within the content in different mobile sets,
- the functionality of settings in different mobile sets,

For this purpose, ten patients who had purchased their CPAP devices from two sales companies in Mashhad, were literate, and had smartphones to work with effectively were selected. They used the application for two weeks. Patients’ comments and issues they faced while using the application were recorded in the form of unstructured interviews. They were then provided to the technical team after more scrutiny for further development of the system. The detail of the functionality evaluation process is provided in Appendix 1B.

#### Evaluation of doctor’s portal and selling companies’ portal

As the system development was done via the agile, recursive, and incremental method, during the project development process, different versions of the doctor’s portal and the selling companies’ portal were continuously made available to doctors and sales companies. After the evaluation, their comments were obtained through unstructured interviews and applied as far as possible by the technical team. This process continued until the end of the system design and final approval by the users of the two portals.

## Results

### System architecture

Following the stakeholders’ input, the research team (including researchers and system developers) concluded that a patient interface needs to be designed for smartphones because of its easy use and access. The operator and doctor interfaces need to be made to suit PCs corresponding to the data protection legislation.

**Fig. 2:**
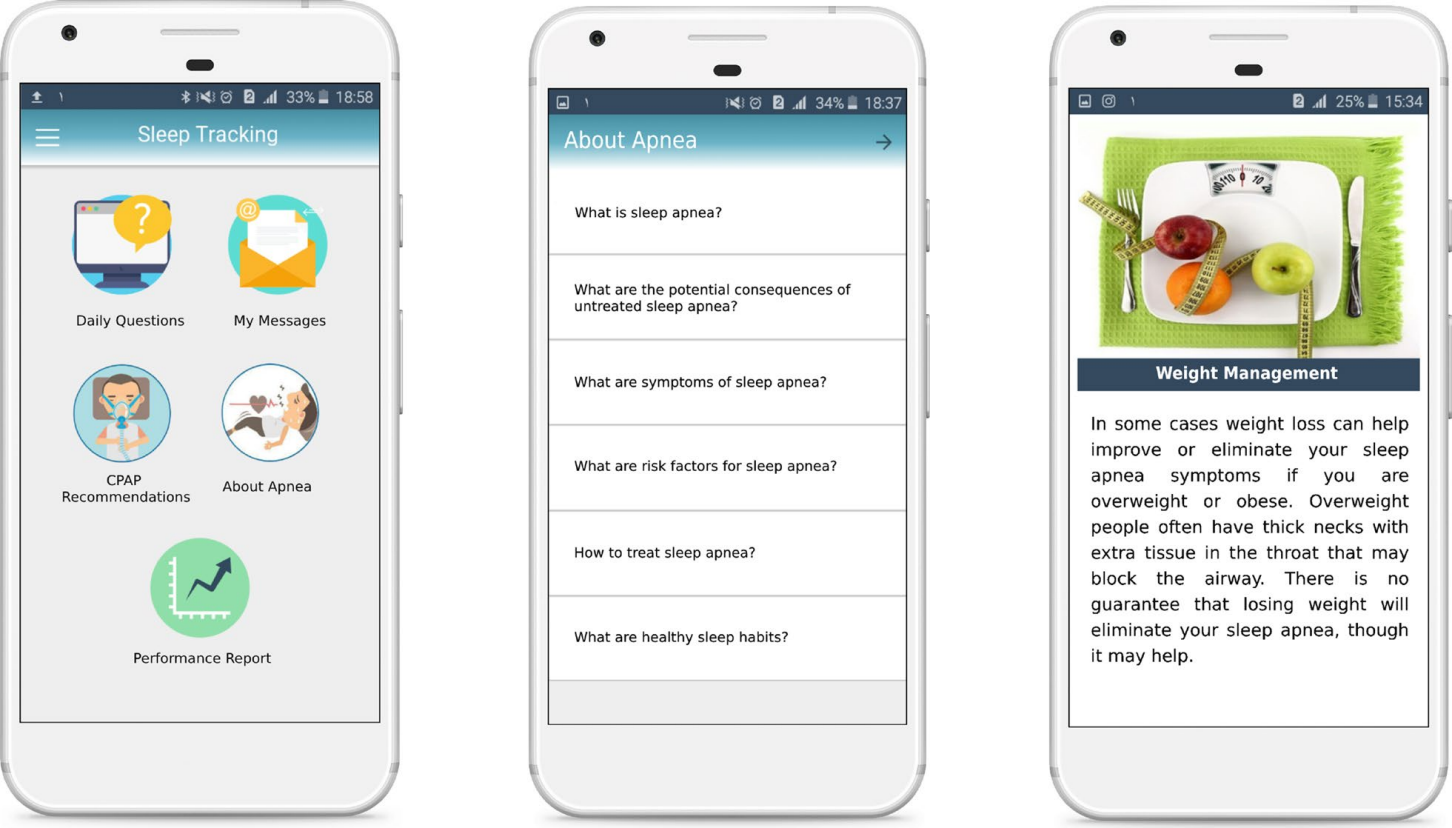
The main page of application and the pages of educational content

**Fig. 3:**
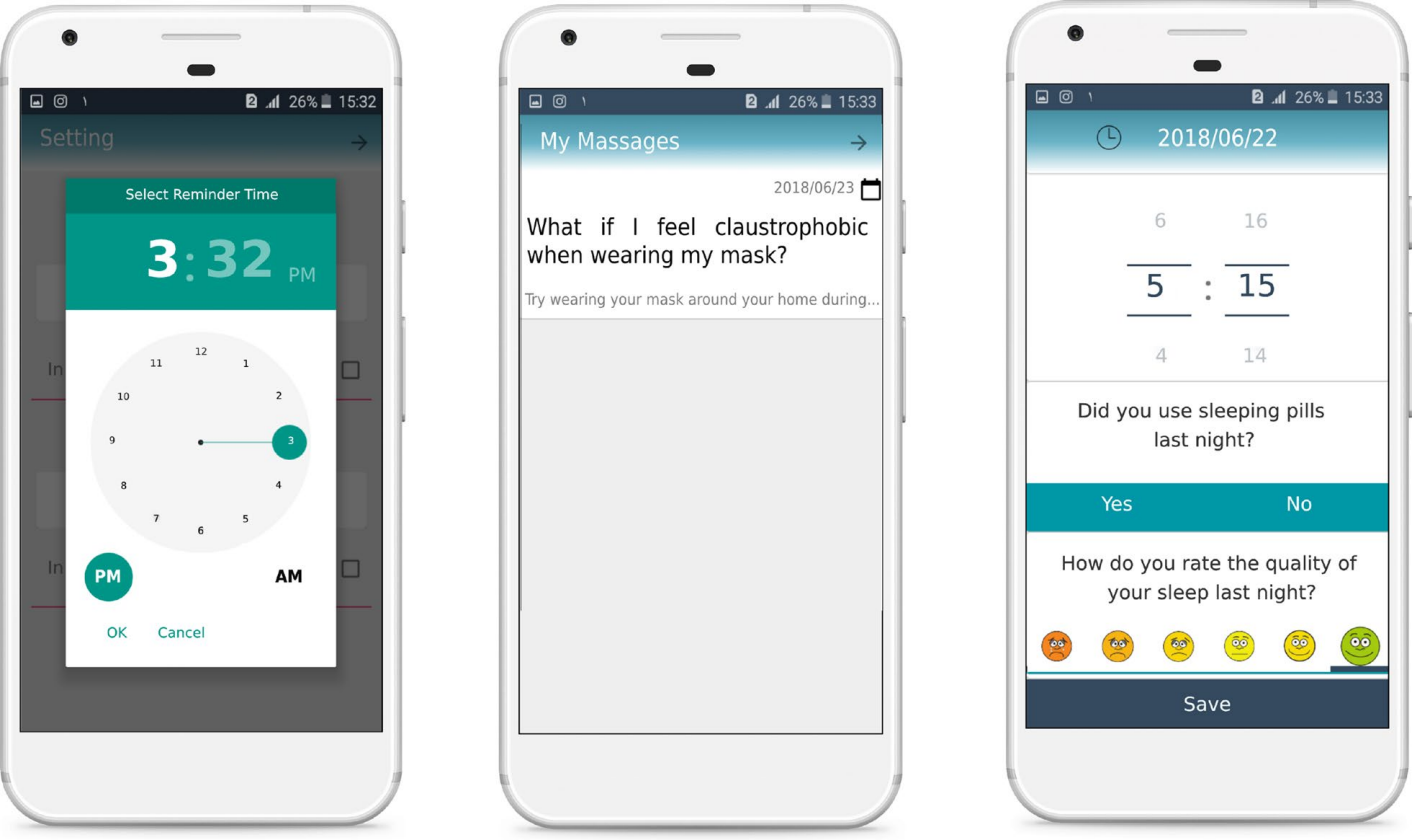
The pages of daily queries, daily messages and setting notification time

**Fig. 4:**
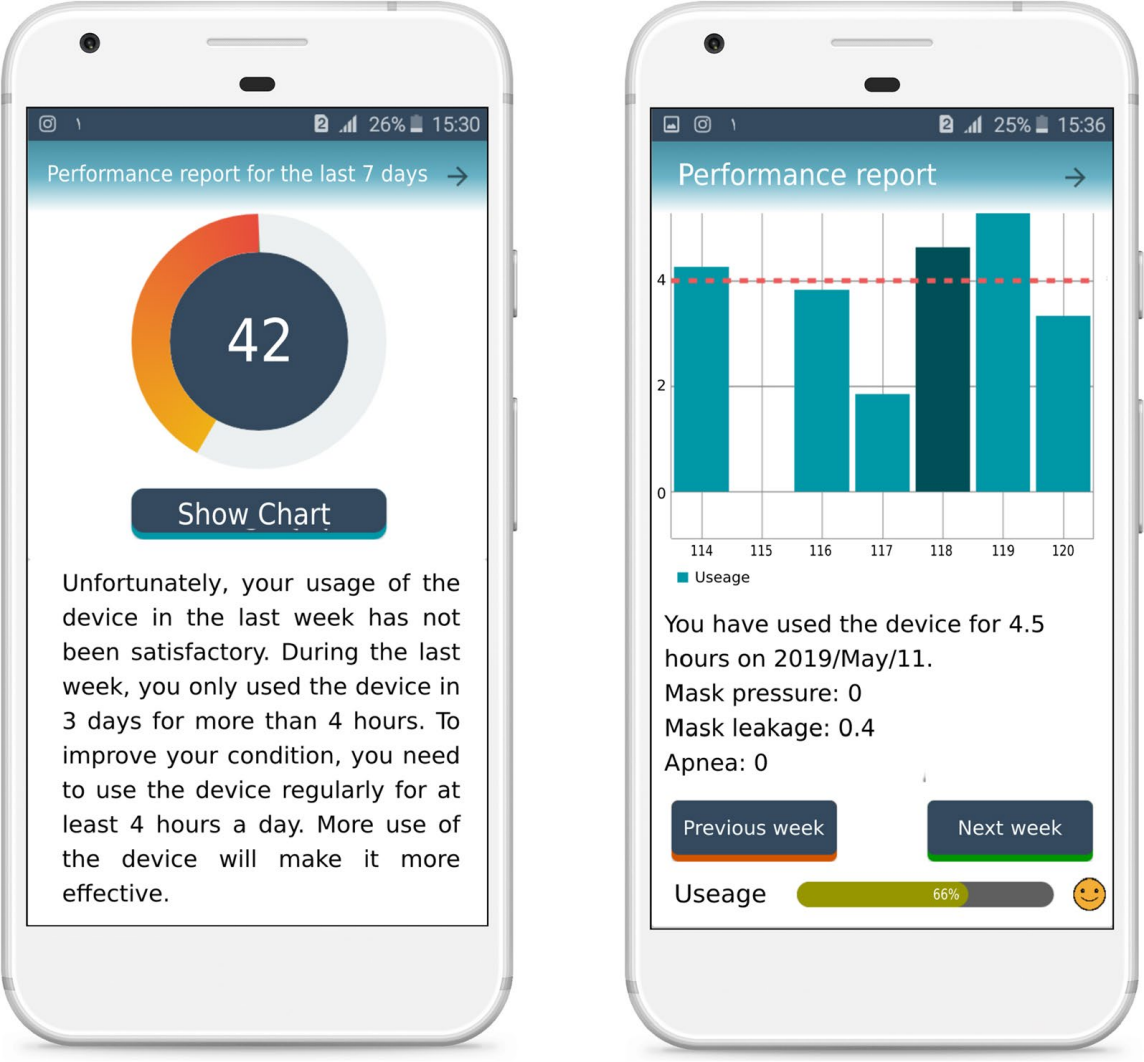
The pages of performance report including the feedback message and the diagram showing the extent to which the device is used

**Fig. 5:**
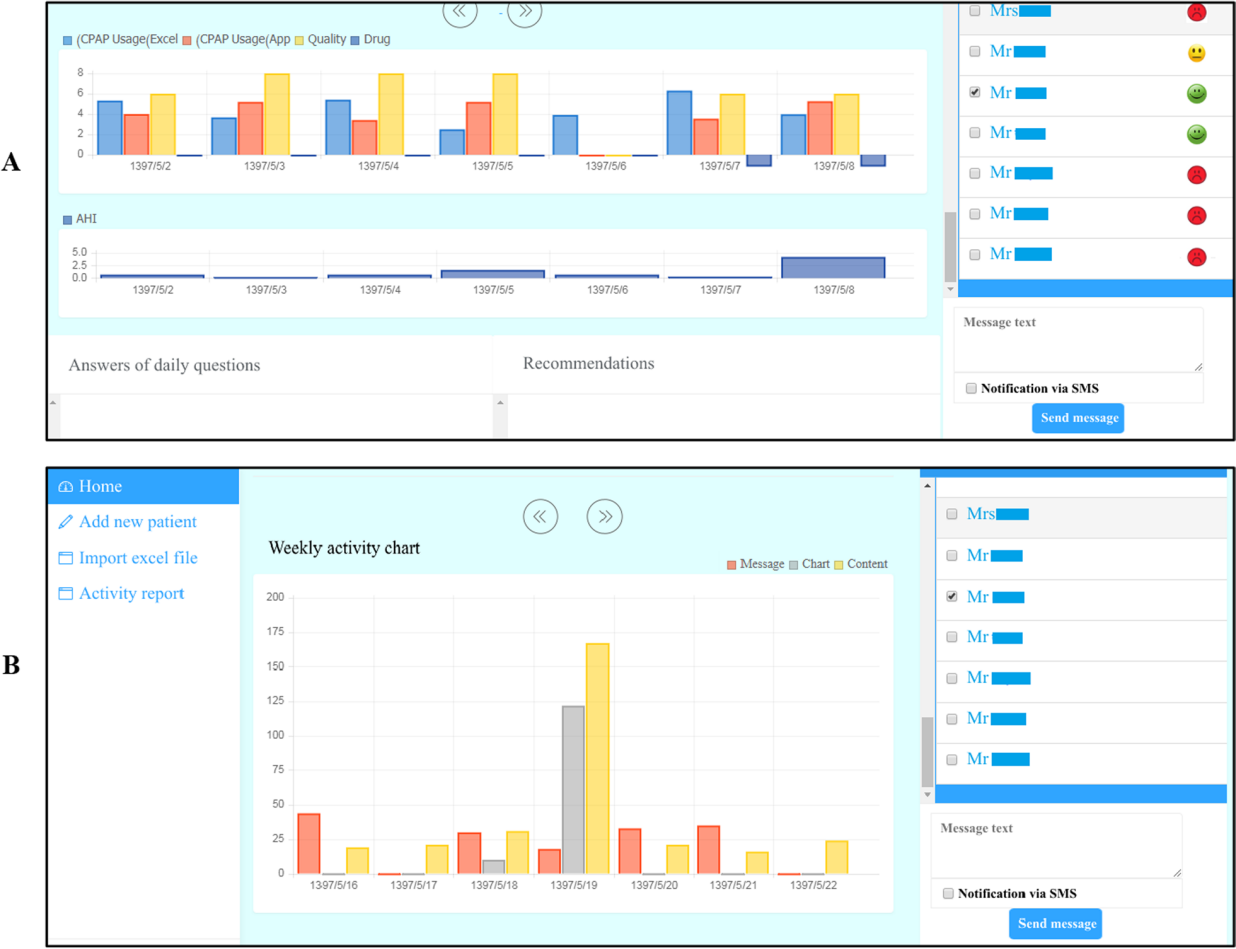
**A** Doctor’s portal, **B** Operator’s portal

- *Patient’s application* The application was provided to the patient with the capabilities of educating them, reporting on performance, sending and receiving information, and showing notifications. The main parts of the patient’s application were educational content (Fig. 2), daily queries, daily messages (Fig. 3), and reports of performances (Fig. 4). Also, The checklist of barriers to device use was displayed to non-adherent patients (Table 3). The marked reasons were sent to the doctor’s portal to control and possible recommendations. A sample of feedback displayed on the performance report page is presented in Table 2. The detail of each part of the patient’s application is described in Appendix 1C.
- *Doctor’s portal* The doctor’s portal was designed for the visiting doctor. Patient’s answers to daily questions, actual use of the device, adherence status, reasons for non-adherence, the capability of sending messages to patients, and taking notes for future visits were the main features of the doctor’s portal (Fig. 5A).
- *Operator’s portal* An operator’s portal was made available to the selling companies. Recording patients’ demographic and clinical information, uploading the CPAP data, and activating the notifications were the main features of the operator’s portal (Fig. 5B).

**Table 2:** A sample of feedback messages

Score^*^	Messages
100	Congratulations: your performance was excellent last week!
	You used the device more than 4 h a day last week
	Using the device regularly, having enough physical activities and a good diet will definitely pull you closer to your goals
71	The extent to which you used the device last week was very good!
	Last week, you used the device more than 4 h a day for 5 days
	A regular use of CPAP helps to remove many symptoms of apnea. Moreover, managing many background diseases such as hypertension, diabetes and cardiovascular diseases is facilitated
	Using the device regularly, having enough physical activities and a good diet will definitely pull you closer to your goals
14	Unfortunately, you did not use the device properly last week
	In order to improve the disease, you need to use the devise regularly at least 4 h in 24 h. The more regularly you use the device, the more effective it becomes
	See details on mask leakage, device pressure, and frequency of respiratory intervals in different days. To solve probable issues, use the educational content provided. If the problem is not solved, consult a doctor or the company staff
	The following list is probably the reasons why you did not use the device regularly last week. Please read it carefully and mark each that is true for you. At the end, press the “next” key

^*^Score: number of days the device was used more than 4 h/total number of day

**Table 3:** The checklist of barriers to device use

#	Barriers
1	When using the device, my mouth, throat or nose feels dry or cold. I feel my nose is blocked and cannot breathe properly, So, I often breathe through mouth
2	I feel uneasy wearing the mask (e.g. it irritates my face or causes perspiration)
3	I need to go to the bathroom at night
4	The noise of device disturbs me and my spouse
5	Air leakage from edges is disturbing
6	Using the device makes me nervous and I feel suffocated
7	The air pressed into my nose disturbs me
8	Often, I fall asleep before using the device
9	While asleep, I take off the mask
10	I have nightmares
11	Despite using the device, I keep snoring. Sometimes I feel suffocated as I feel something jerks into my throat
12	I have gritting teeth
13	I have a running nose
14	The water stuck in tubes and the mask is not feeling good
15	At night, I feel anxious or depressed and sometimes sleepless
16	At night, I cough
17	The device disrupts my sleeping position. Sometimes I prefer to sleep one side or face-down but the tube is too short or gets tangled
18	I see no use of it
19	I was travelling

*Data sending software* due to the lack of capability of automatic sending of CPAP data to the central system, a facilitator software was designed and offered to patients on a CD along with instructions on how to use and install it.

#### Technical aspects

The system development was done through an agile, recursive, and incremental method. Changes suggested by users during the project development and afterward were applied after examining the feasibility and practicality. The software was launched for the client through HTML/CSS/JavaScript and the server databank was MySQL 4.5.2. The programming language was PHP and the data transfer occurred through the web services developed in PHP, too, and in the JSON format. Mobile application programming was done natively using Android Studio version 3. The.csv file data were extracted from the server and stored in the MySQL database server. When the request was sent from the application, the patient’s data were sent in JSON format. All educational files were made available to users in an offline mode. The store and forward mechanism was used to send daily queries to the server.

### System evaluation

#### Usability testing of patient access module (patient’s application)

**Fig. 6:**
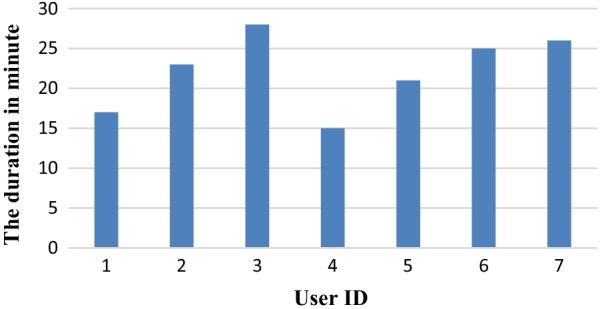
The usability testing time for each user

In usability testing, a think-aloud protocol analysis was done with seven patients (six men and one woman) whose average age was 44 ± 10.2 years. The mean time of the evaluation process was 22 ± 4.7 min. The time it took for each user to do the evaluation is presented in Fig. 6.

The total number of issues found with usability by users in the evaluation process was 18, with an average of 2.5 issues per user.

The layout issues of the system constituent parts were 8 in number and comprised 44.5% of the total issues (the most frequent). The most important changes were:

1. Location of the list of problems on the performance report page, the display type, and the selection mode,
2. Color of the CPAP usage diagram,
3. Details and headings of the CPAP usage diagram axes,
4. Display daily messages,
5. Font size.

The next rank belonged to vocabulary issues with five cases (28%). One issue of this type was the unintelligibility of the names of buttons. To tackle this problem, the names of the buttons were changed in a way that better fitted the target action.

As for the data entry issues, four cases were found (22%). One of the most critical problems was entering data for daily queries. To solve this issue, the type of data entry was changed. Besides, to set the notification sending time, in the settings, the type of data entry changed according to the users’ suggestions.

**Fig. 7:**
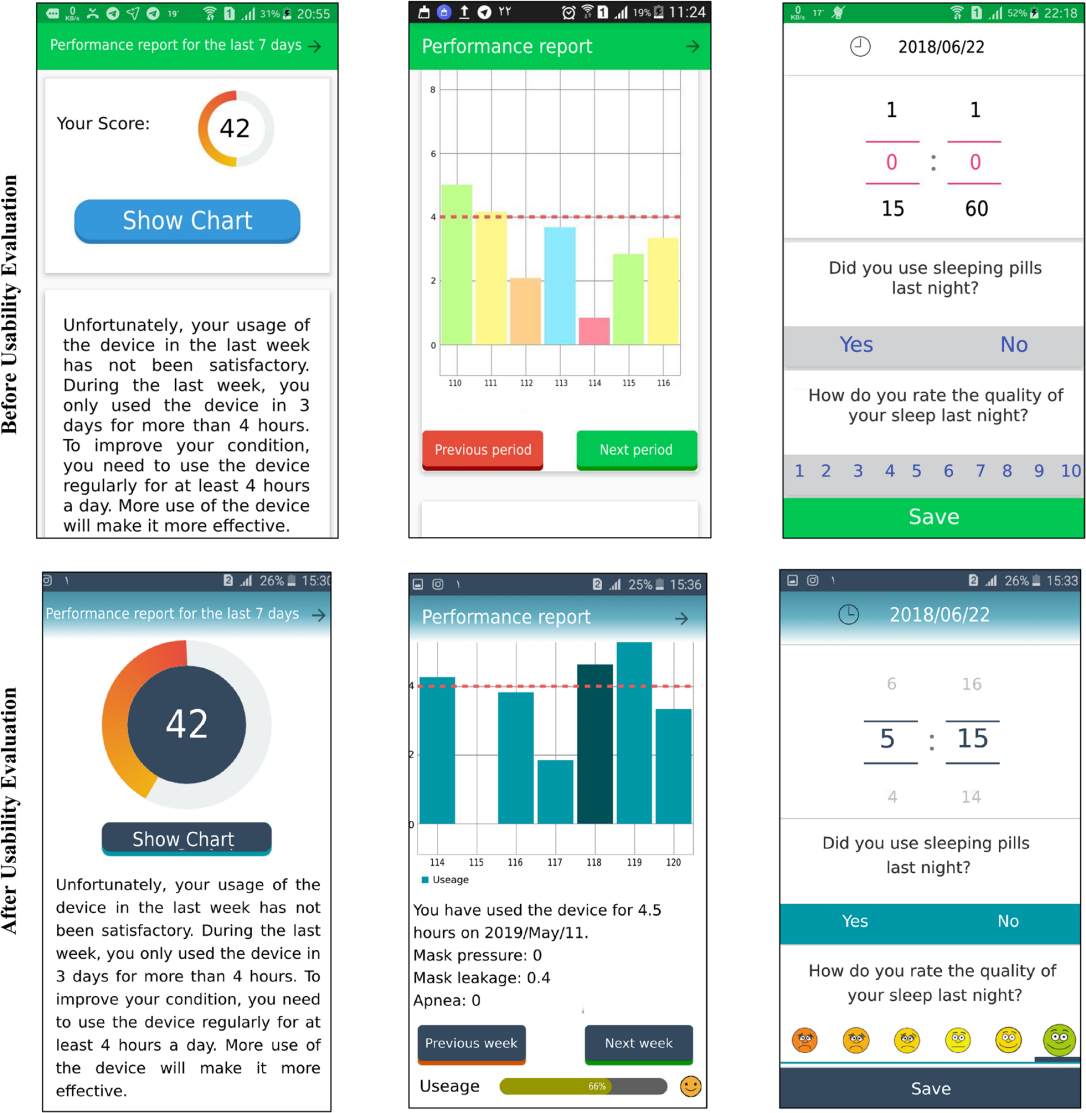
A sample of changes in application pages before and after usability testing

As for issues with comprehensiveness, there was only 1 case (5.5%) about touching the columns of the performance diagram that indicated the device’s daily use (including the extent of use, amount of leakage in the mask, number of apnea, device pressure). As we could find no technical solution to this issue, adequate instructions were provided to patients when installing the application. Some changes to the application after usability testing are shown in Fig. 7.

#### Functionality testing of patient’s application

The main problems detected in functionality testing were: not working properly on devices in languages other than English, disruption in the installation process, no link between the portals and the application, and improper playing of videos on some mobile sets with small screens. Once these problems were found, they were solved by the technical team.

## Discussion

The present research described the design, development, and evaluation of a patient telemonitoring system for those afflicted with sleep apnea undergoing CPAP therapy. To our knowledge, there has been almost no research to use a participatory analysis, design, and evaluation approach to incorporate stakeholder inputs, evidence, and behavior change theories and techniques in developing a patient monitoring system for patients with OSA using PAP machines. Also, this is the first study on the Iranian population that differs from previous studies regarding cultural, economic, and social factors. So, it would be a complementary study to previous ones in other societies.

Obstructive sleep apnea is a chronic disease that needs to be treated through the long-term use of PAP devices. To manage the disease, many groups are involved including doctors, selling companies, and the patients. Due to the significance of interaction among these three key elements, the system was designed in a way that physicians and companies could provide their services to the patient as far as possible. To this aim, three parts were integrated into the system: the patient’s application, doctor’s portal, and operator’s portal.

A key feature of the system was the recurrence of educational content through daily messages and the creation of a link in different parts of the patient’s application for easier access to the internal content. As the related literature indicated, recurrent exposure to educational content can improve treatment adherence. Some other research findings using daily text messages (e.g., SMS) to change health-related behaviors showed that these interventions were truly effective in changing behaviors in patients and promoting preventive behaviors and self-care in chronic diseases [45]. It is also noteworthy that though education is essential for a basic knowledge of behavior change, information and recommendations might be inadequate for significant changes in an individual’s behavior [45–48].

Some parts of the application content were using educational videos that helped patients to use and maintain the device, giving feedback on patients’ performance followed by suggestions to non-adherent patients to solve the existing problems, and sending daily messages that reminded the side effects of irregular use of the device or not using it at all. These could pave the way for changing the patient’s behavior. Of note is that the application content was based on the socio-cognitive theory and the correlation between the constituent constructs of this theory (i.e., knowledge, perceived risk, outcome expectancy, self-efficacy) and CPAP adherence proved in many studies [40–43].

As some users of the application are elderly, we attempted to facilitate the use of the application as much as possible for them. As it was required to enter data in the application or send it to receive feedback, the older users needed more help and support to use the application. However, younger users used the application more freely and independently. As the new technologies such as mobile-based health can be challenging to the elderly, we tried to use larger font sizes and shorter sentences that were easier to follow. Other relevant attempts included no use of specialized terms in content, straightforward design of the interface, display of adequate explanations upon entering the application, and the possibility of making changes to settings.

Another advantage of the system was monitoring patients’ performance from far. Doctor’s monitoring makes patients feel more responsible for their conditions and more motivated to use the device in order not to disappoint the doctor. This point has been raised in other studies, too, that eliciting patients’ feeling of responsibility in comparison to educational-only interventions can significantly affect the adherence behavior [39].

Wireless telemonitoring in the initial stages of CPAP treatment can significantly reduce the nursing time compared to usual care. Prompt troubleshooting during CPAP therapy can increase patients’ adherence to treatment. Telemonitoring can save nursing sources and lead to regular follow-up and adequate support. Consequently, treatment adherence is increased [49]. Moreover, for patients with physical disabilities, who cannot move easily or live farther than the sleeping centers, telemonitoring can save the patient’s and healthcare providers’ time due to tele-settings and tele-troubleshooting.

### Study limitations, strengths, and future directions

There are several strengths in the present study. first, the development process was accompanied by the stakeholders collaborating closely. These included patients, healthcare providers, researchers, system developers, and patient representatives. Thus, this research ensured that content and software functionalities were included that were both clear and meaningful to patients and healthcare providers. This process can help to increase the potential effectiveness of the target intervention. Another strength was that in the design and development of the system, different methods were used for data collection to meet the users’ needs and make the best use of existing capabilities. Moreover, using the behavior change techniques in preparing content and system capabilities probably enhances the effectiveness in changing the patient’s behavior of using the CPAP device. Finally, evaluating system usability and functionality was done after designing and developing the system which led to the recognition and removal of system defects for further research.

This study has several limitations. The sample size was limited which negatively affected the usability testing. However, 3–5 participants are enough for usability testing [50]. Another limitation was that no clinical outcome was explored in this research as here the aim was to design, develop and evaluate the software in terms of usability and functionality to solve potential problems in the forthcoming body of research. It is suggested that the effectiveness of the telemonitoring system be investigated as a mobile health intervention compared to usual methods and the results are provided for higher-level decision-makers.

## Conclusion

Proper management of OSA needs a long-term use of CPAP devices and subsequently, constant clinical and technical support. The present research elaborated on the participatory analysis, design, and evaluation approach, including stakeholder inputs, evidence, and behavior change theories and techniques when developing the patient monitoring system for patients with OSA who used CPAP machines. While the system efficacy remains to be evaluated, content and system development were based on the need analysis of stakeholders. So it has the potential to improve CPAP adherence in patients with OSA.

## Data Availability

The datasets used and/or analyzed during the current study are available from the corresponding author on reasonable request.

## Appendix 1

### A: Usability testing of the patient access module (patient application)

To test the usability of the patient application, the think-aloud protocol was used, which is a user-based method widely used. Think-aloud is an empirical method based on the observation of system performance in time [44]. The think-aloud method aims to collect data related to users’ cognitive growth in working with the system. In this method, users are asked to state out loud whatever they see, think about, and feel, as well as the questions that arise in their mind or their decisions [51]. In this method, the user states his/her thoughts, activities, and observations while working with the system verbally.

Participants The participants in this study included three groups: users, facilitators, and a technical supporter. The users were 7 patients using the CPAP. They had no knowledge or experience of the Roya telemonitoring system. For the evaluation process, each user was accompanied by a facilitator, who did not interfere in the evaluation process and only reminded them of speaking loudly when they faced a problem in the process. Facilitators were among medical informatics experts with adequate knowledge and experience of testing usability. A software engineer was also present during the evaluation process as the technical support just in case.

Time and place of evaluation: The evaluation process was followed in a room of a sleep test clinic within a peaceful and light environment. There were a table, two chairs and a tablet connected to the internet. To do the evaluation process, such equipment as the Screen Recorder was installed on the tablet computer to record the user’s interaction with the system and a microphone to record the user’s voice. To conduct the evaluation process, a scenario was developed based on the tasks and duties of the Roya telemonitoring system.

Evaluation process Before evaluation, the think-aloud protocol was taught to the users. In 10 min, they were instructed on how to express their thoughts, feelings, and decisions in detail. Moreover, the issues with conducting the precise evaluation process were reminded to facilitators. They were asked not to make any interference in the evaluation process for the user and not to impose any personal comment. They were supposed to constantly encourage the user to speak aloud while thinking. When the evaluation was done, the users were asked to give suggestions for the improvement of system performance.

*Data analysis* When the evaluations were completed, all the recorded documents were analyzed by the facilitators. To categorize problems, the method suggested by Haak et al. [52] was used, according to which the problems were categorized in 4 groups:

- Problems with the system layout (e.g., the user cannot find the key to go to the next page)
- Problems with terminology (e.g., the user cannot understand the meaning of some items)
- Problems with data entry (e.g., the user does not know how to enter data in a specific field)
- Problems with comprehensiveness (e.g., the user feels that the system guideline is not comprehensive)

### B: Functionality testing of patient access module (patient’s application)

10 patients who had purchased their device from two sales companies in Mashhad and were literate and had smartphones to work with effectively were selected. They set prior appointments with a sleep specialist and visited him. The doctor had a full examination of the patient’s conditions and the extent to which s/he used the device. After visiting the doctor, the purpose of the research was explained to the patients who involved evaluating the functionality of the system and asking for their opinions to make improvements in the system. If they were willing to participate, they registered in the portal and had the application installed on their smartphones. The data concerning the extent to which the device was used was derived from CPAP and uploaded in an Excel file into the system. When the application was installed, the patient could log into the application with a username and password and receive the required instructions on using the application from the researcher.

Patients followed up for two weeks. Those who received the application sent the information stored in the CPAP memory card through emails once a week (overall two times during the evaluation course) and received feedback on it on their mobile application. When there was no possibility of emailing the information on the memory card by the patient or his/her company, the data sending software, which was already designed by the researchers to match the devices of each brand, were provided to the patient on a CD along with its instructions on how to use them. If the patient did not send the weekly report (at the end of the first and second week), a reminder message was automatically sent to him/her.

After the follow-up, the patients were contacted to remind them of the second visit and referral to the clinic. In the second visit, patients’ comments and issues they faced while using the application were recorded in the form of unstructured interviews and were then provided for the technical team after more scrutiny for further development of the system. The main points taken into account in the evaluation of the functionality of the application were:

- Successful installation of the application on smartphones each with a different version of Android operating system
- Successful transfer of data on CPAP device from the operator’s portal to the application
- Successful transfer of responses to daily questions and user’s problems from the application to operator’s portal
- Accurate display of charts and content in different mobile sets
- Accurate display of videos integrated within the content in different mobile sets
- Accurate functionality of settings in different mobile sets

### C: Patient access module (patient’s application)

- *Educational content* included information about apnea and treatment with CPAP. This information was presented in textual and audio-visual modes. To facilitate the patient’s access to the educational content, the prepared content was presented in short text chunks with specific headings (Fig. 2).
- *Daily queries* asked to what extent the device was used the previous night, whether sleeping pills were taken or not and the quality of sleep the night before. If the question about taking sleeping pills was answered as No three times consecutively (for three consecutive days), it was omitted. Following the time set by the user for the Q&A part of the application, a notification was sent by the device at the same time. The user was guided to daily questions by touching the notification message. The mechanism of sending messages to the server was “store and forward”. Thus, the answers to questions were saved in the memory according to their date and then transferred to the server once connected to the internet (Fig. 3).
- *Daily messages* corresponded to the application content and contain problems with the device, side effects, significance of disease, and so on. Daily, one to two messages were sent to patients. According to the time set by the user in the application to receive daily messages, a message was sent along with a notification at each time. The messages were saved in the inbox to make later checking possible. They were saved chronologically from the newest to oldest in the inbox (Fig. 3).
- *Report of performance* involves feedback on the extent to which the device was used in the past week and the whole period of time (Fig. 4). A sample of feedback is presented in Table 2.

According to the related literature, feedback can only have positive effects on health when it aims to support and guide in the right direction [53]. Accordingly, in the report on performance section, after receiving the required feedback on the frequency of using the device during the past week, if one was recognized as non-adherent based on the pre-defined criteria, a checklist was presented of the probable reasons for not using the device regularly (Table 3). It is noteworthy that this list was made based on the qualitative content analysis of texts, patients’ interviews, and the specialists’ comments. For each item within the list, several solutions were thought of. This solution was based on the educational content of the application offered in different words. The patient used the inserted hyperlink to access the internal content of the application at the end of each solution for further information. Finally, the patient was asked to register the selected items in the system in to inform the physician. This allows the doctor to intervene and send further advice in situations where the patient's problem is recurring.
